# Supplementary material for: Perceived fear and exercise difficulty in patients with migraine and their association with psychosocial factors: a cross-sectional study
Source: PeerJ. 2025 May 12;13:e19342. doi: 10.7717/peerj.19342 (PMC12085118; doi:10.7717/peerj.19342)
Supplement: Supplemental Information 1 [file peerj-13-19342-s001.pdf]

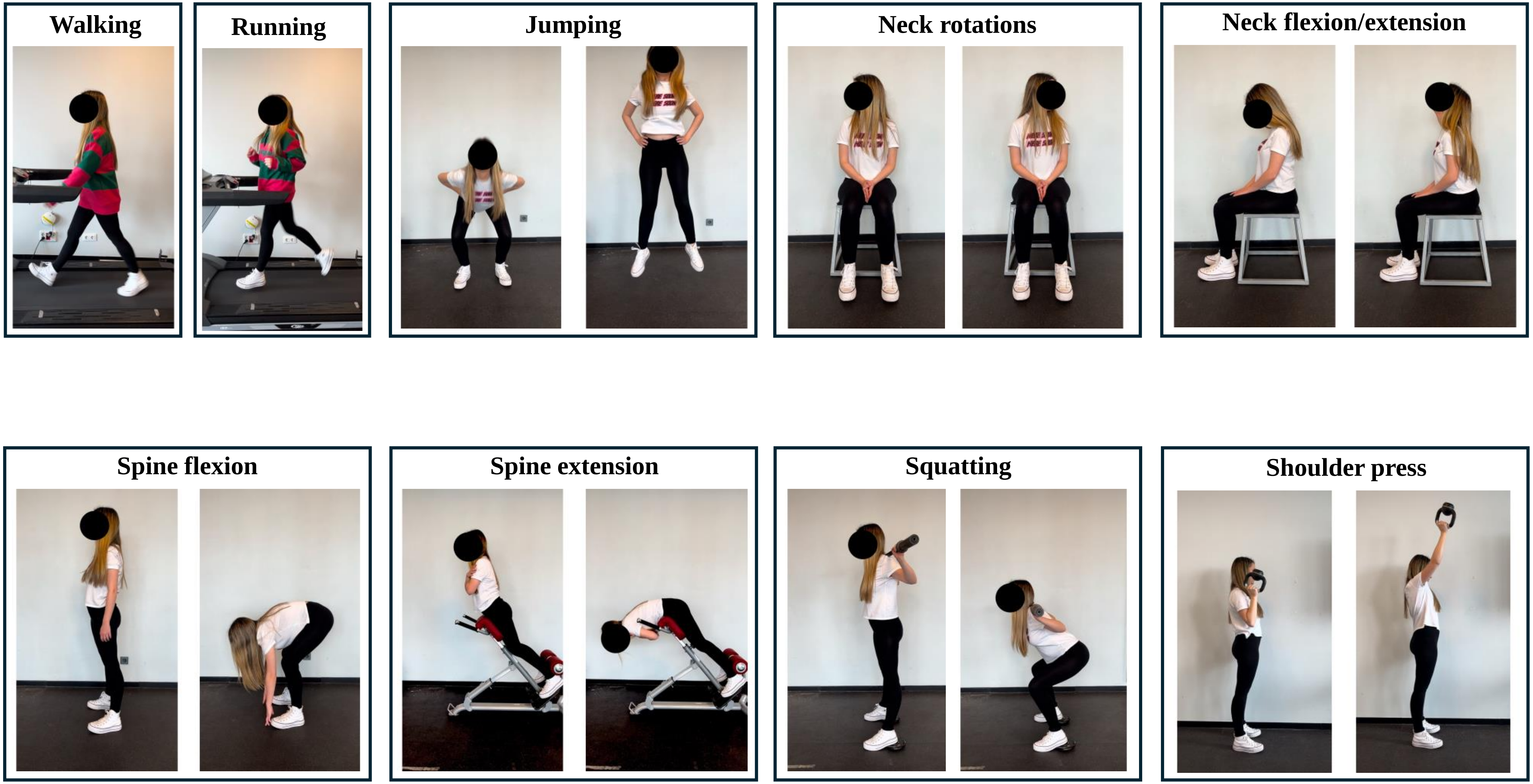
Walking
Running
Jumping
Neck rotations
Neck flexion/extension
Spine flexion
Spine extension
Squatting
Shoulder press
