# Supplementary material for: Perceived fear and exercise difficulty in patients with migraine and their association with psychosocial factors: a cross-sectional study
Source: PeerJ. 2025 May 12;13:e19342. doi: 10.7717/peerj.19342 (PMC12085118; doi:10.7717/peerj.19342)
Supplement: Supplemental Information 2 [file peerj-13-19342-s002.docx]

**Supplementary Material S2.** FAB reliability results for scale and elements.

| **Scale reliability** | | | | |
| --- | --- | --- | --- | --- |
|  | **Mean** | **SD** | **Cronbach’s α** | **McDonald’s ω** |
| Scale | 1.9 | 1.2 | 0.87 | 0.88 |

| **Element reliability** | | | | |
| --- | --- | --- | --- | --- |
|  |  |  | **If the element was discarded** | |
|  | **Mean** | **SD** | **Cronbach’s α** | **McDonald’s ω** |
| Item 1 | 0.45 | 1.2 | 0.87 | 0.88 |
| Item 2 | 3.6 | 2.06 | 0.87 | 0.88 |
| Item 3 | 3.13 | 2.33 | 0.86 | 0.87 |
| Item 4 | 3.04 | 2.27 | 0.86 | 0.87 |
| Item 5 | 1.07 | 1.9 | 0.87 | 0.87 |
| Item 6 | 2.94 | 2.4 | 0.86 | 0.87 |
| Item 7 | 0.35 | 1.19 | 0.87 | 0.87 |
| Item 8 | 1.48 | 1.85 | 0.86 | 0.86 |
| Item 9 | 3.1 | 2.23 | 0.86 | 0.86 |
| Item 10 | 1.79 | 2.15 | 0.85 | 0.86 |
| Item 11 | 2.18 | 2.18 | 0.86 | 0.86 |
| Item 12 | 2.34 | 2.27 | 0.86 | 0.87 |
| Item 13 | 0.66 | 1.68 | 0.86 | 0.87 |
| Item 14 | 0.46 | 1.26 | 0.86 | 0.86 |
