# Supplementary figures and images for: Perceived fear and exercise difficulty in patients with migraine and their association with psychosocial factors: a cross-sectional study
Source: PeerJ. 2025 May 12;13:e19342. doi: 10.7717/peerj.19342 (PMC12085118; doi:10.7717/peerj.19342)

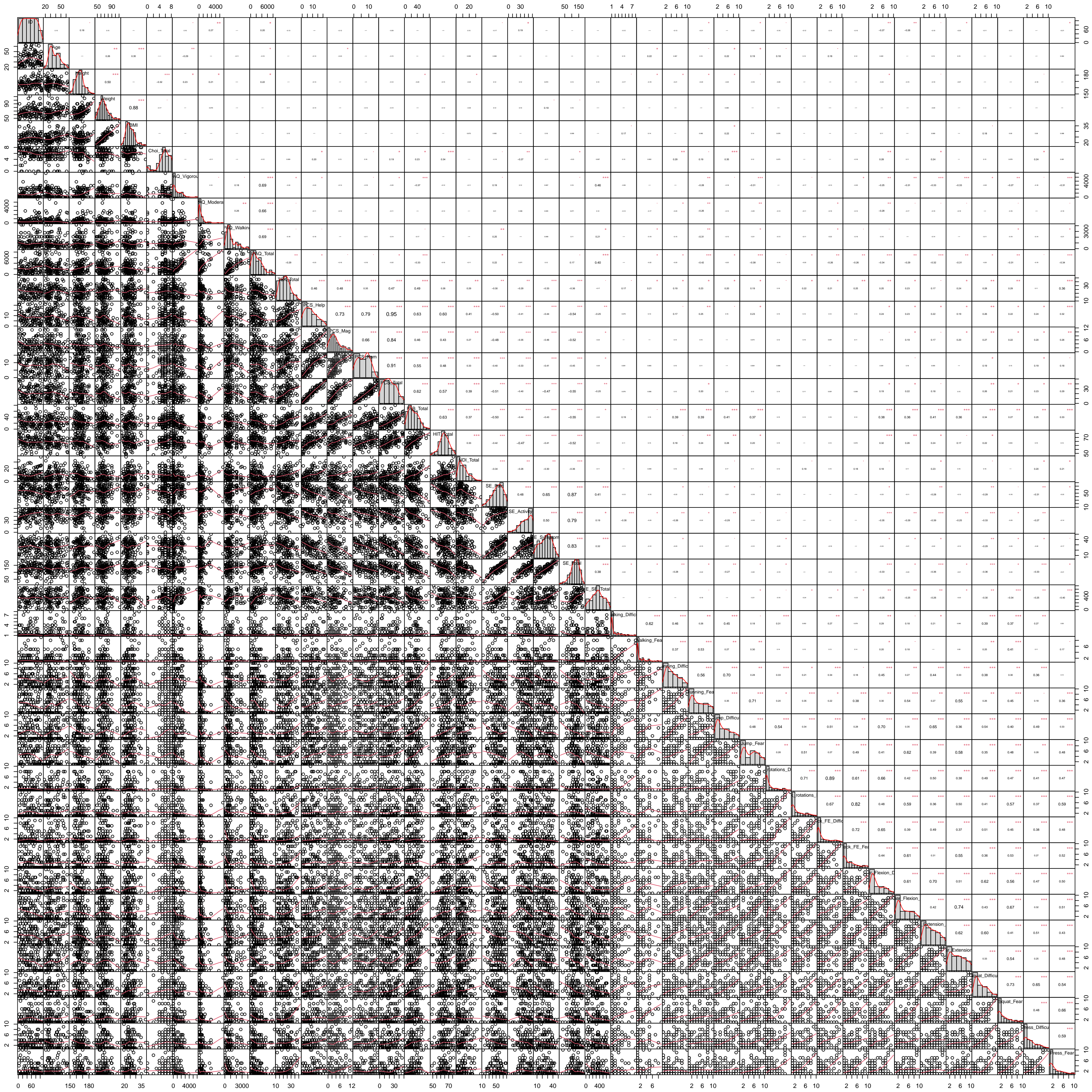

Supplement: Supplemental Information 3 [file peerj-13-19342-s003.pdf]

a)

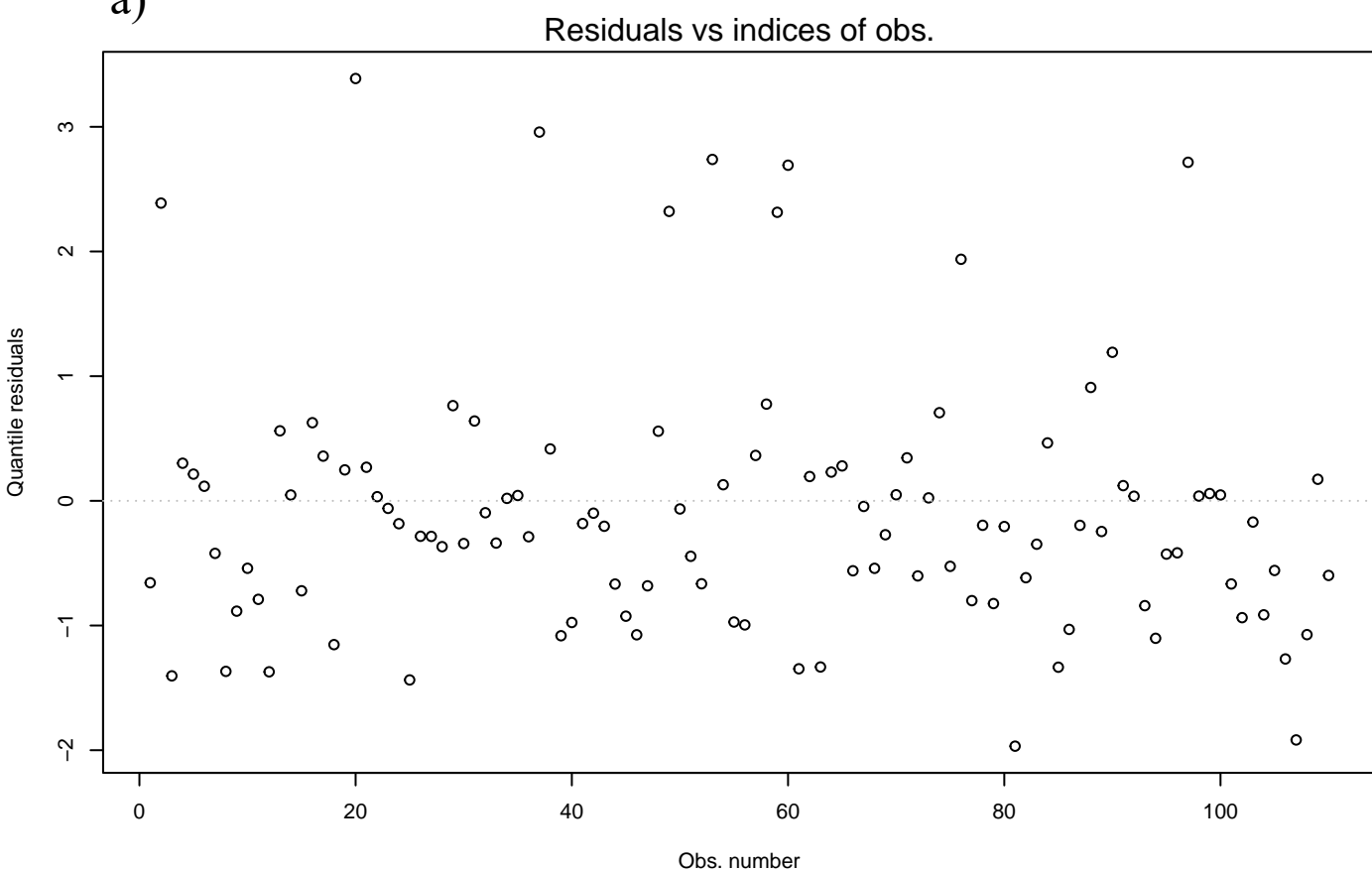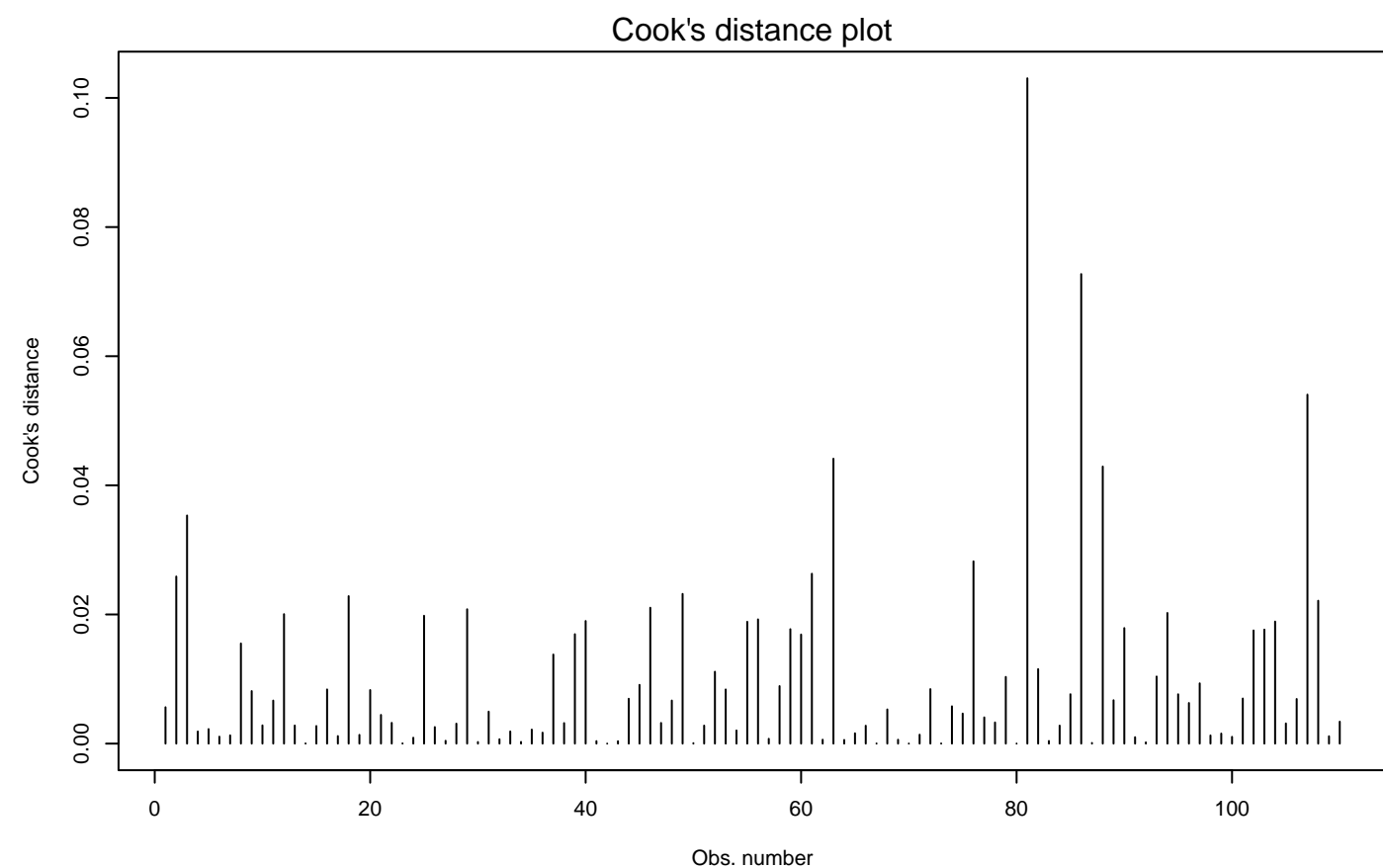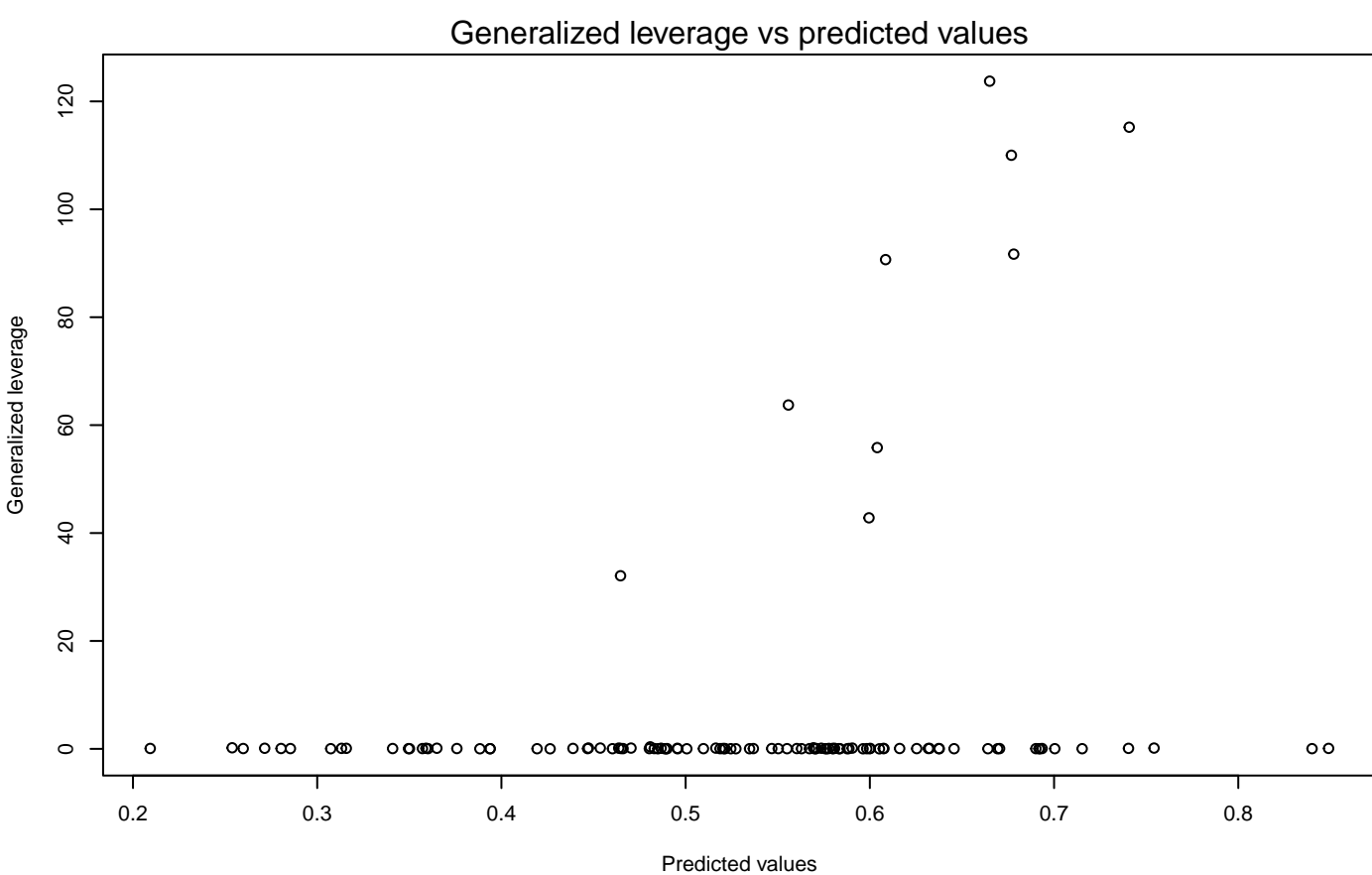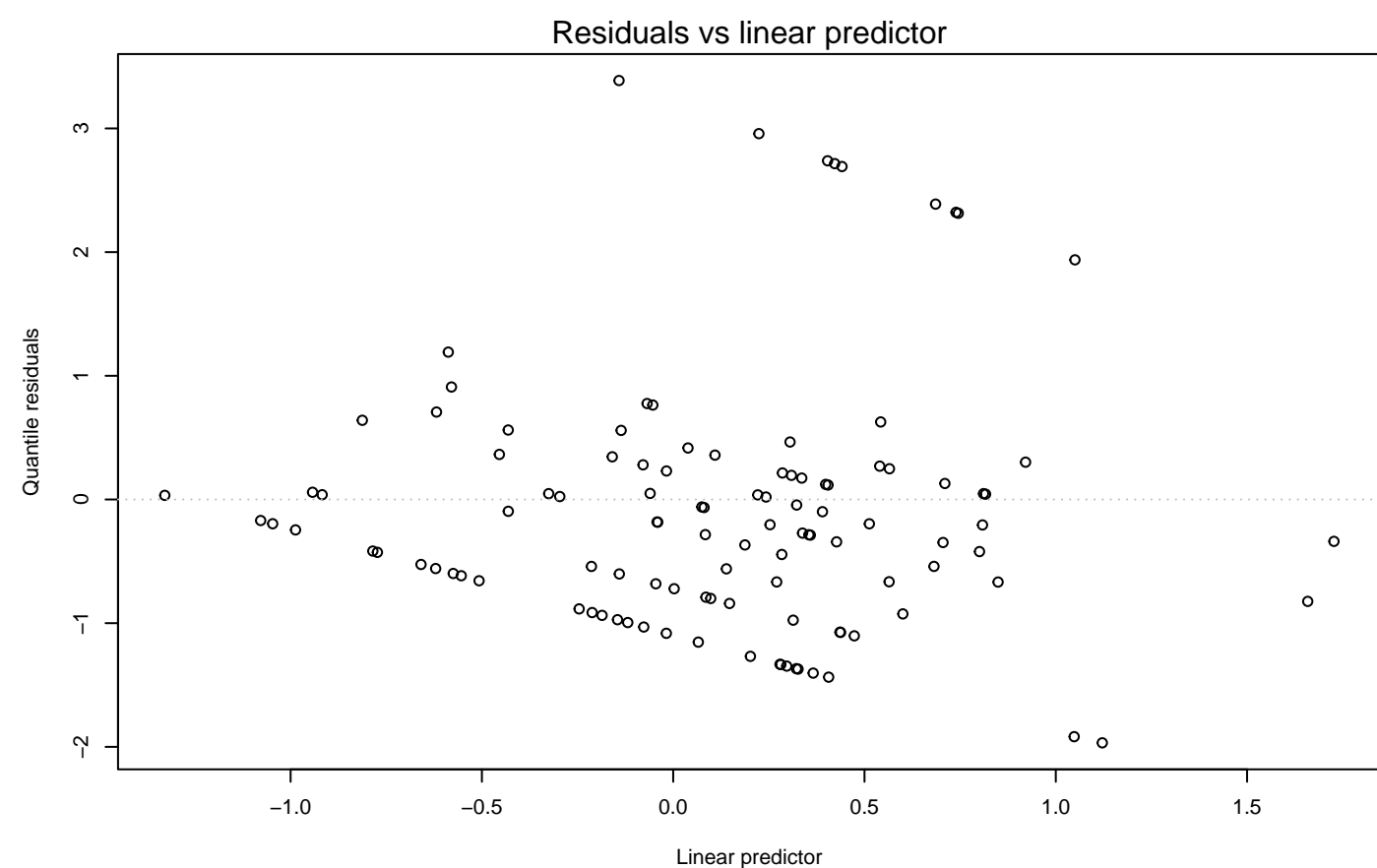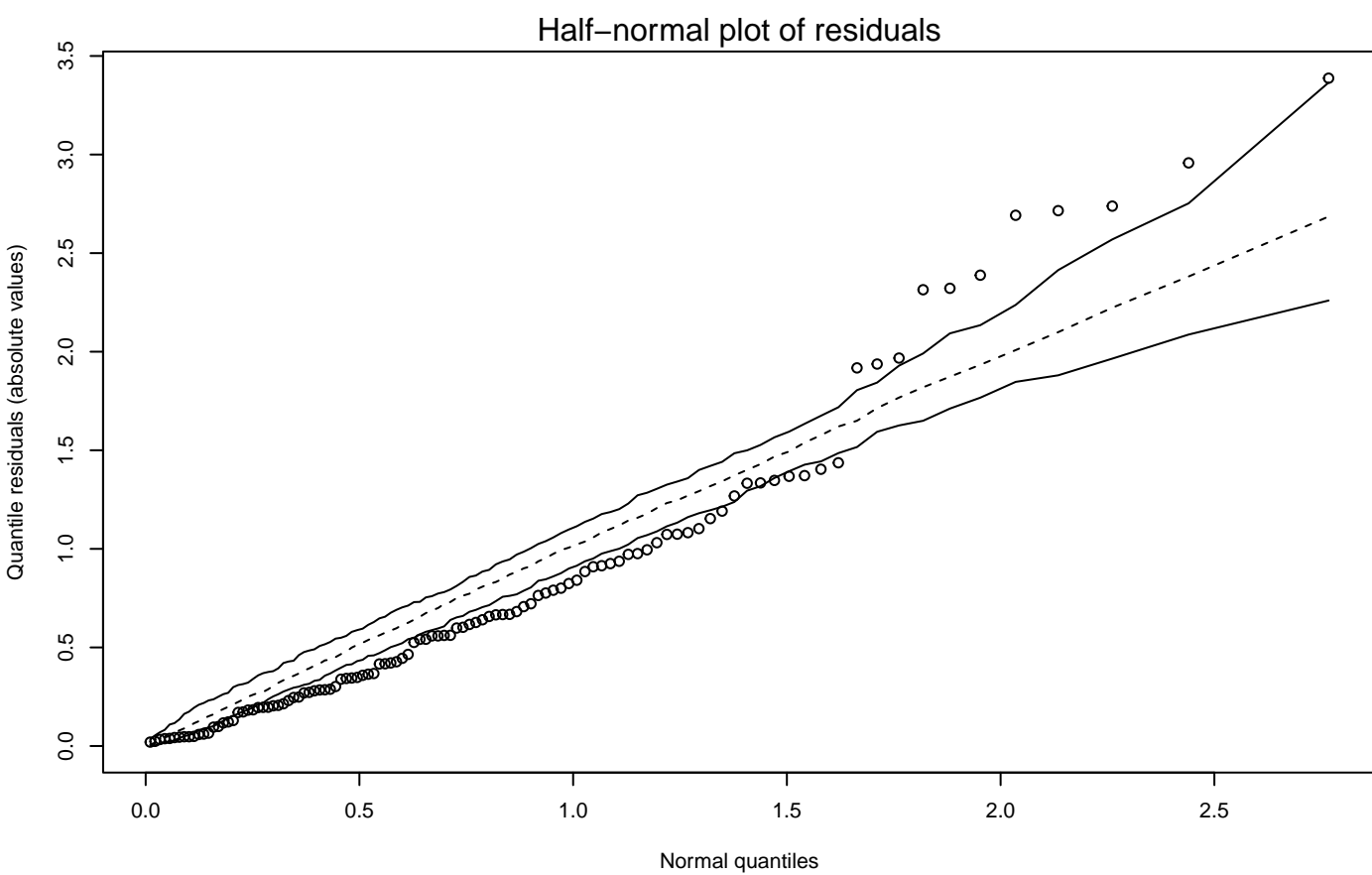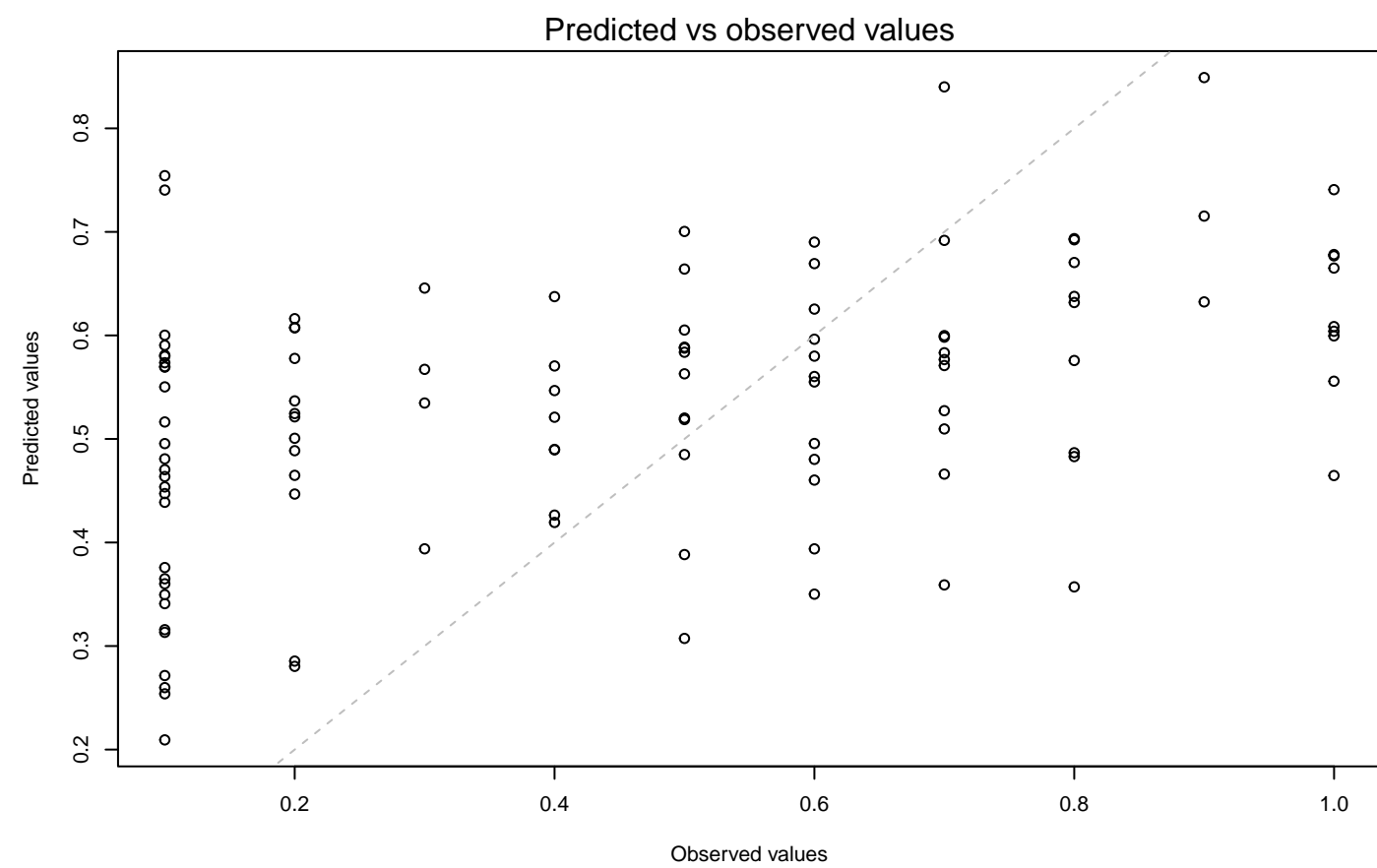

b)

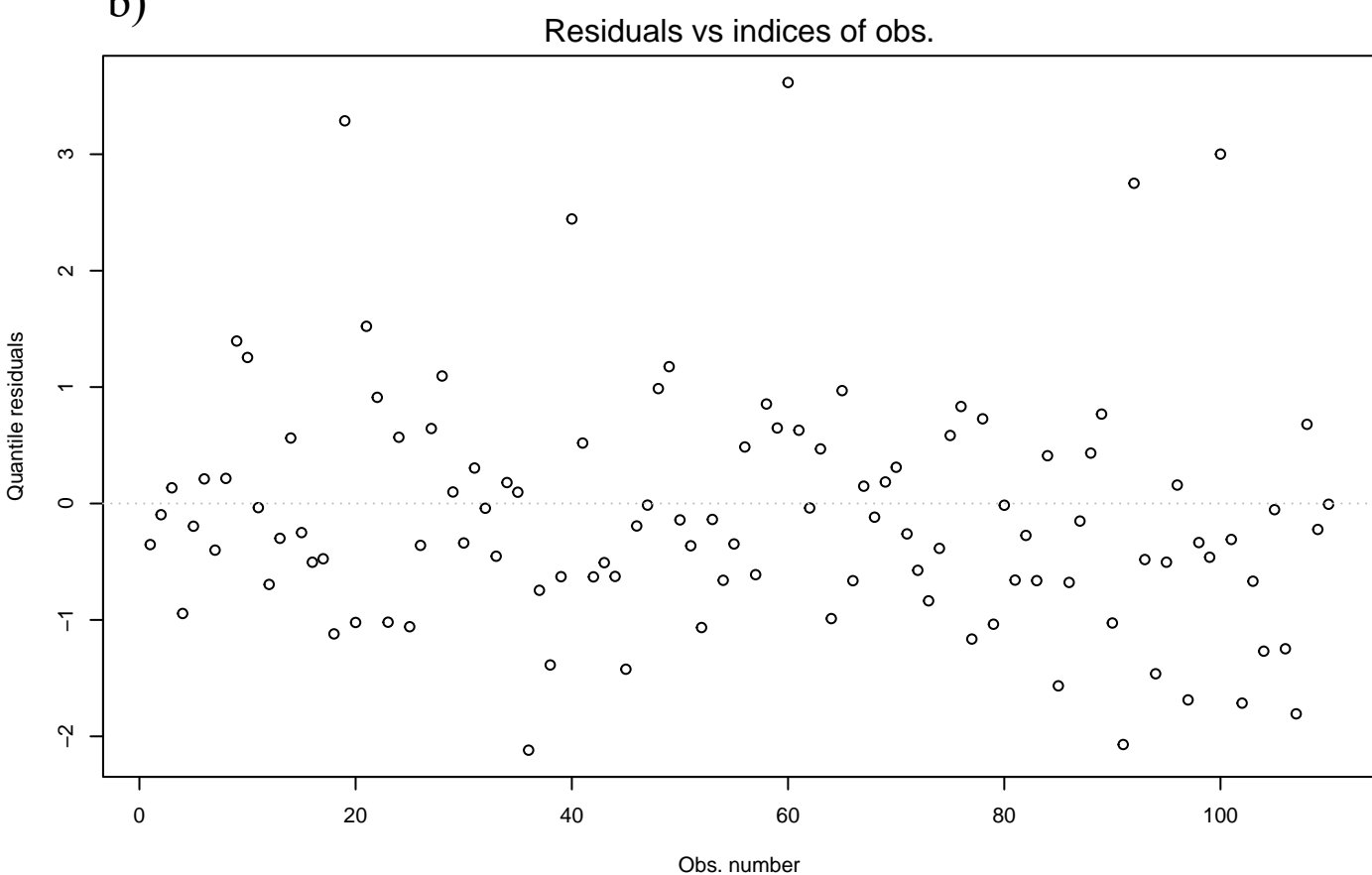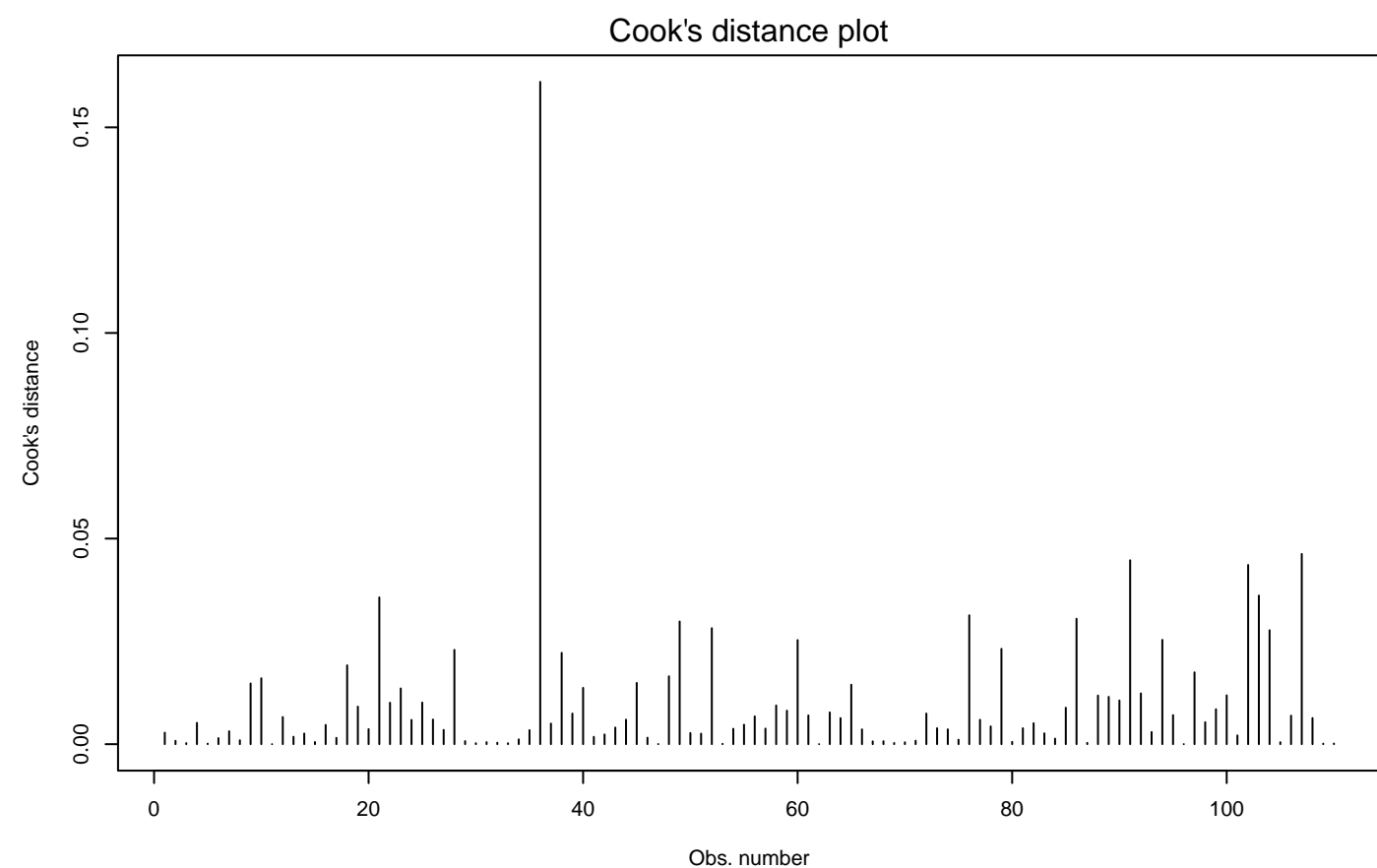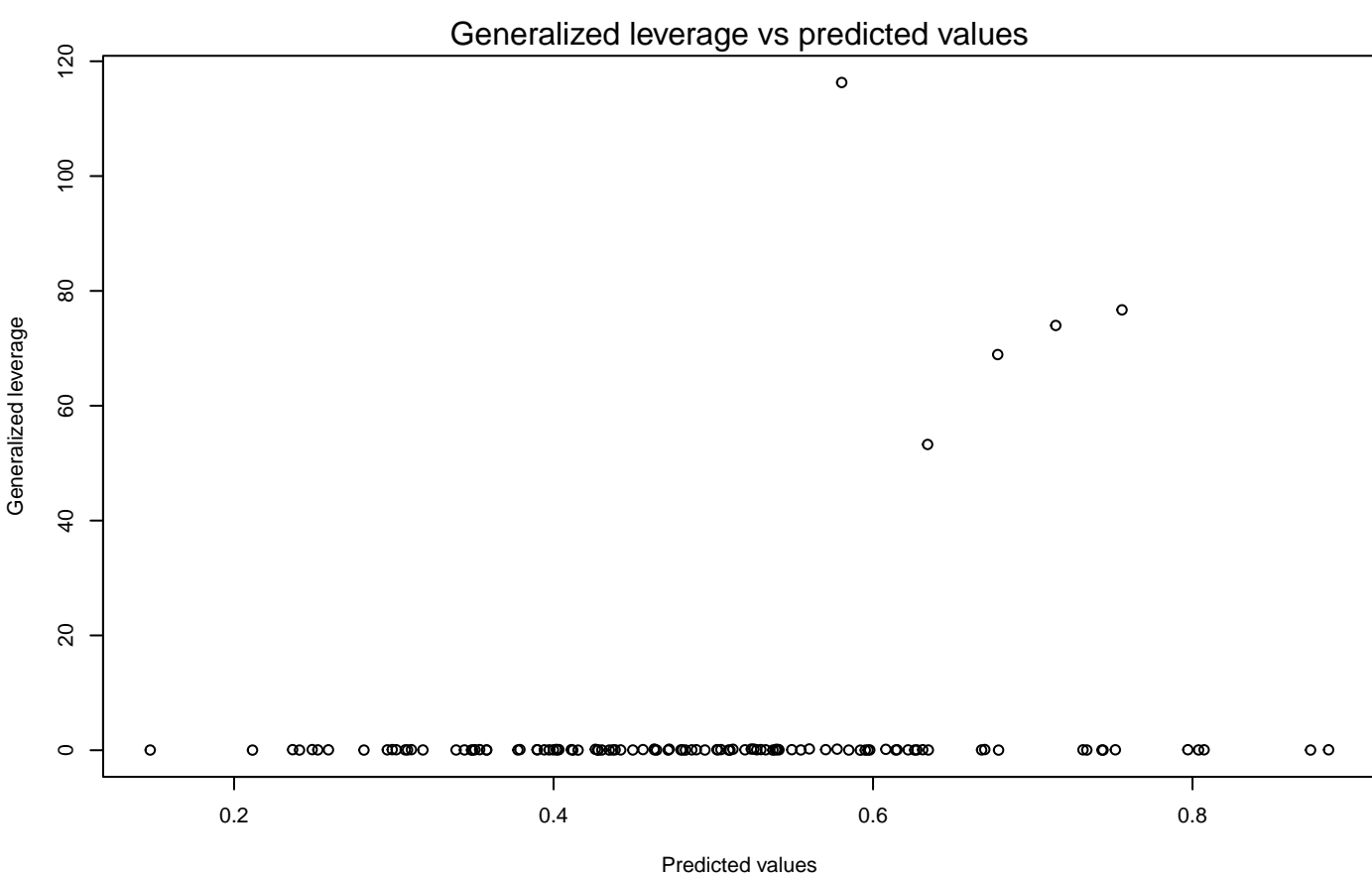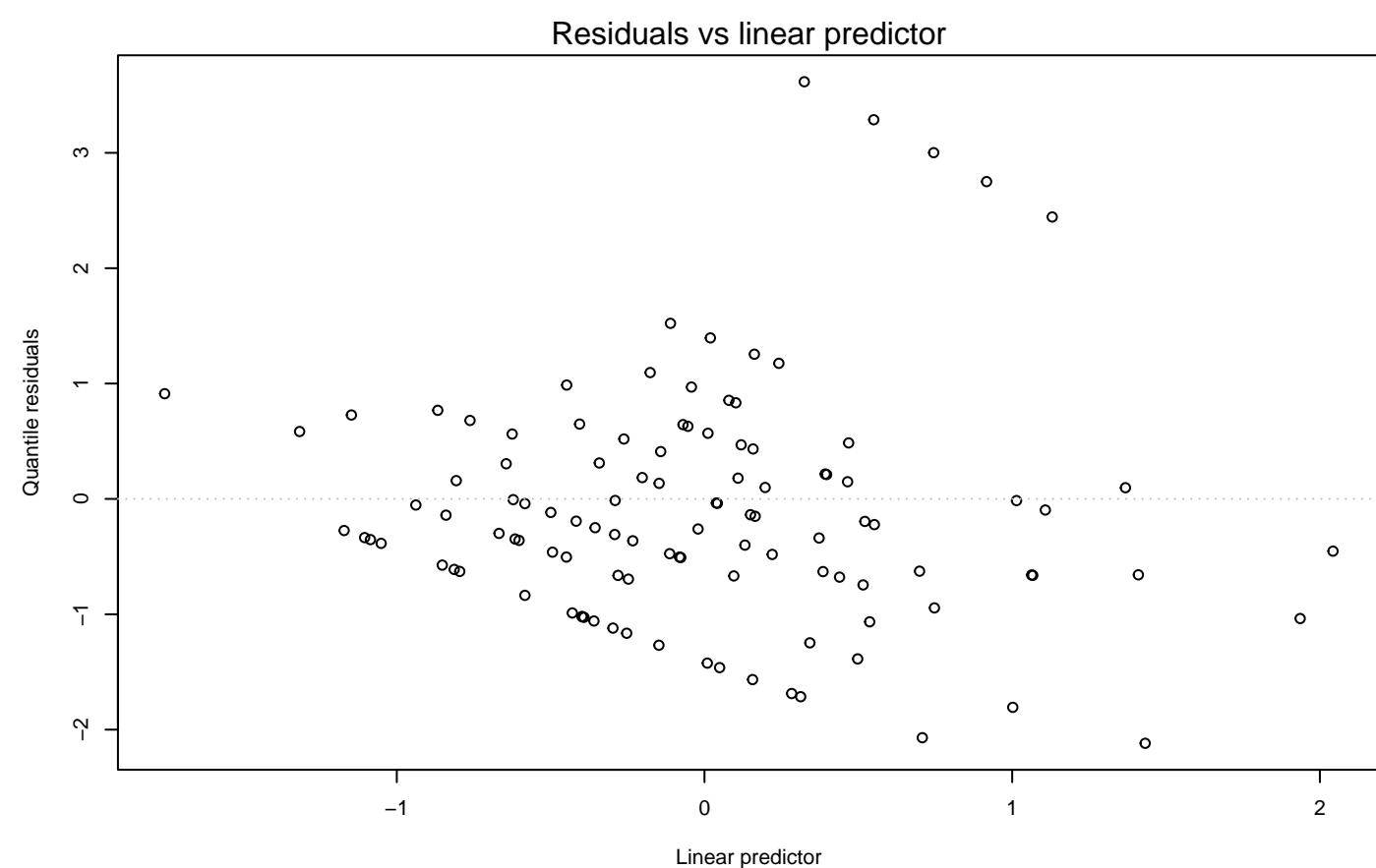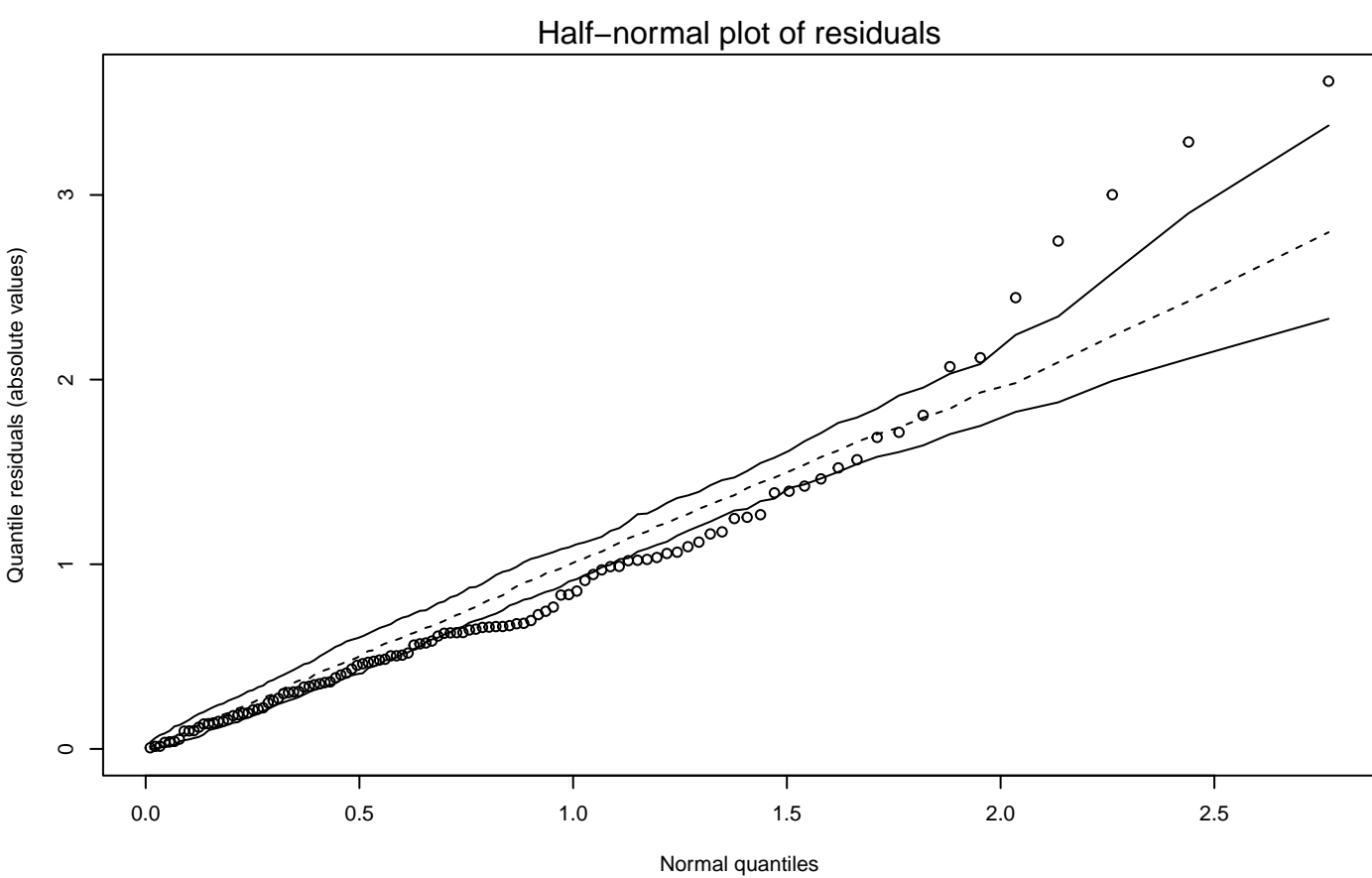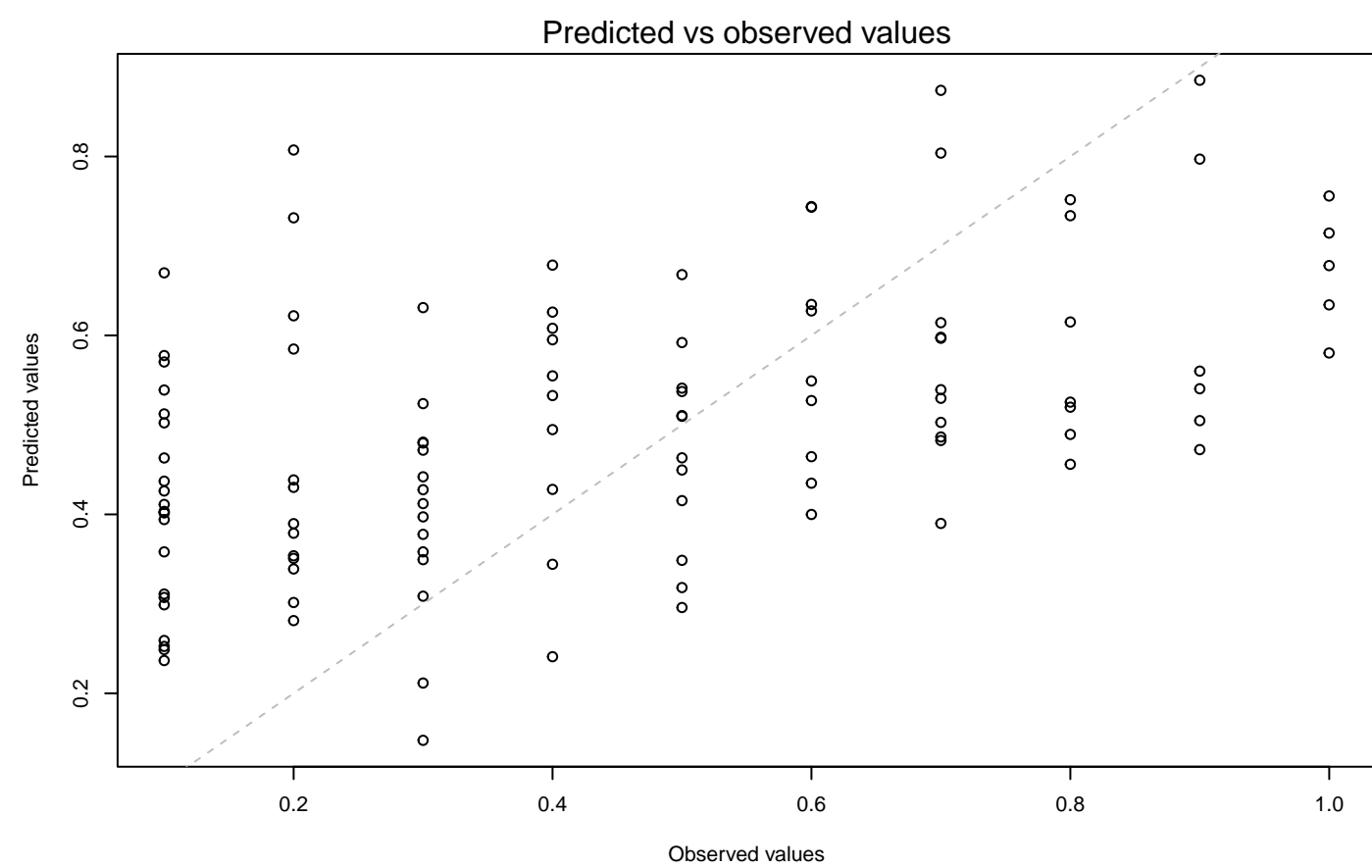

Supplement: Supplemental Information 4 [file peerj-13-19342-s004.pdf]
